# A Qualitative Exploration of Peer Supporters' Experiences of Undertaking a Co‐Produced Mental Health and Emotional Well‐Being Training Programme

**DOI:** 10.1111/hex.70084

**Published:** 2024-10-29

**Authors:** Laura Kane, Lauren Walker, Judith Eberhardt, Robert M. Portman, Emma‐Lily Proctor, Hannah Poulter, Catherine O'Neill

**Affiliations:** ^1^ Department of Psychology, School of Social Sciences, Humanities & Law Teesside University Middlesbrough UK

**Keywords:** co‐produced training, emotional well‐being, peer support, peer support training programmes, recovery, social support network

## Abstract

**Introduction:**

Peer supporters play a crucial role in mental health and support services, but their own mental health and emotional well‐being are often neglected by themselves, and, frequently, their organisations. Here, we report findings from a qualitative interview study of peer supporters who completed a co‐produced emotional well‐being training programme.

**Method:**

Ten semi‐structured interviews with peer supporters working in the North East of England were conducted to explore their experiences of the training programme.

**Results:**

Thematic analysis of the data produced three overarching themes. In Theme 1, ‘Increasing psychological preparedness and identifying self‐care and coping strategies’, we found that peer supporters improved their knowledge of how to manage sensitive topics such as aggression and suicide and felt more confident in their peer support roles resultantly. In Theme 2, ‘It's good to know you're not alone’, peer supporters discussed their experience of loneliness in their roles, and as a consequence realised their own need for peer support to help maintain their well‐being. Theme 3, ‘Toward the future: next steps’, encapsulated peer supporters' willingness to continue their role development and to create a peer support network to continue to obtain mutual support.

**Conclusion:**

Our findings emphasise the perceived emotional well‐being benefits of a co‐produced peer supporter training programme. Participants highlighted the need for co‐produced training programmes that are (1) emotion‐focussed, (2) provide access to other peer supporters and (3) provide future avenues for a peer supporter network of mutual support and professional development activities and training opportunities.

**Patient or Public Contribution:**

Individuals with lived experience of mental ill health and peer support were consulted in the development of interview questions and provided feedback on the finalised themes to ensure the analysis and interpretations were congruent with their experiences.

## 1 Introduction

Peer support involves people with lived experience of mental health conditions, disability or adverse experiences who guide others towards recovery [1]. This guidance provides hope by modelling successful, ongoing recovery [2]. In recent years, peer support provision has increased due to policy changes aiming to embed recovery‐oriented practices [3, 4] and due to the positive outcomes associated with peer support. Davidson et al. [5] categorise peer support into three types: (1) naturally occurring and informal peer support, (2) working or volunteering in peer‐led or peer‐focused programmes or services and (3) peer support within traditional health settings.

Each type has been associated with various positive outcomes. Naturally occurring and informal peer support often fosters community and mutual aid, increasing hopefulness and self‐efficacy [6, 7]. Peer‐led or peer‐focused programmes, where individuals work or volunteer, have been linked to improvements in self‐esteem, condition management and reduced hospital admissions [8, 9]. Within traditional health settings, peer support services enhance client–provider relationships and improve outcomes, such as treatment retention and satisfaction [10, 11, 12].

Moreover, peer support's effectiveness extends to online programmes, which can reflect any of the three types. For example, online peer support for higher education students has shown significant benefits. Drysdale et al. [13] and Grégoire et al. [14] report that students in online peer support programmes self‐reported reduced psychological inflexibility, stress, anxiety and depression, along with increased psychological flexibility and well‐being.

Whilst peer supporters report many benefits of their role, including improved self‐esteem, self‐determination, and sense of purpose [15], they also face significant challenges [16]. One major issue is role ambiguity, as they are often not given clearly defined job roles with defined tasks and duties [17], leading to job dissatisfaction [18, 19]. Furthermore, peer supporters often work in emotionally taxing environments, regularly exposed to grief, trauma and social isolation [20].

Combined with insufficient role‐specific training [21] and a lack of supervision [18], peer supporters struggle to implement and maintain boundaries [22]. They also face challenges in self‐disclosure, needing to balance sharing their lived experience to build rapport against the risks of disclosing too much, which may negatively impact both the peer supporter and the client [19].

To address the challenges faced by peer supporters, researchers have recommended improvements to training programmes. Vandewalle et al. [16] championed the importance of co‐producing training, particularly in managing self‐disclosures. For example, peer supporters should prepare in advance what, and how much, of their lived experience they are willing to share with clients before starting their role [19]. Furthermore, training should include psychiatric rehabilitation models and therapies that follow a humanistic, recovery‐orientated approach [19]. Repper and Watson [23] also emphasised peer supporters' well‐being and the use of experiential methods, such as creating a personal wellness plan and working in pairs during placements to enhance social support.

Drawing upon these principles, previous studies have implemented training programmes to enhance peer support roles, addressing unmet training and role‐specific needs [23, 24, 25, 26, 27, 28]. Some programmes used a co‐production approach [24, 27], and were typically implemented to support participants transitioning to peer support roles within traditional mental health settings [23, 24, 26, 27, 28]. Findings showed that peer supporters benefited from social support, supervision and knowledge acquisition, which increased confidence in their role [24, 26, 27, 28].

Despite these benefits, there are some notable limitations, including participants feeling unprepared for emotional attachments formed with those they support [26] and high attrition and failure‐to‐pass rates [24, 25, 26, 27]. More concerning, some peer supporters experienced recovery relapse during training programmes [25, 28] or did not experience improvements in their mental health, quality of life, hope and self‐efficacy [24]. These findings emphasise the importance of co‐produced training programmes to ensure peer supporters' concerns and needs are addressed, enabling them to prioritise their own well‐being whilst supporting others.

The well‐being of peer supporters is often overlooked, both within their roles [29] and in training interventions [25, 28], which can negatively impact their own recovery. Peer supporters face numerous challenges, often due to insufficient training, that can undermine their recovery maintenance and well‐being [16]. Concurrently, training programmes intended to benefit peer supporters can inadvertently harm their mental health, disrupting recovery and increasing attrition [24, 25, 26, 28].

To address these issues, this study co‐produced a training programme with peer supporters to ensure their training and well‐being needs were met. The first phase, which identified peer supporters' training needs, is reported elsewhere [29]. This paper reports on Phase 2, focusing on peer supporters' experiences with a bespoke training programme in the North East of England. Unlike traditional programmes centred in statutory mental health settings (see [30]), this programme used an asset‐based community development approach. By involving the wider local peer support community, we aimed to identify and address their specific skills and training needs.

This approach mobilises communities by using their strengths or ‘assets’ to create collective, sustainable action [31]. Through asset mapping, we highlighted peer supporter resources across North East England, promoting unity and sustainable support networks [31]. This method aligns with peer support values such as empowerment, mutual support and reciprocity, which emphasise power equality rather than imbalance [32, 33]. Although the value of co‐production is recognised in underserved communities [34], it is often overlooked in peer support research [16]. Our approach ensured that training was tailored to the voluntary sector's peer support community, fostering meaningful and appropriate training. The present study aimed to illustrate the need for co‐production in peer supporter training and to explore how emotional well‐being needs can be addressed through such programmes, based on feedback from the peer support workforce.

## 2 Method

### 2.1 Study Background

The research team and peer supporters in North East England co‐produced a bespoke training programme based on the challenges peer supporters experienced in their roles and their recommendations for overcoming these challenges. Participants enrolled via the Joint Information Systems Committee (JISC) online platform. Since accessibility had previously emerged as a barrier [29], the programme offered flexible training options to enhance accessibility and equity. Participants chose from short (3 days), long (3 weeks) or live‐streamed online (1 day) formats. Table 1 outlines the programme content.

**Table 1:** Topics covered in the training programme.

Training topic
What is peer support?
Taking work home and how to manage this.
Well‐being: Self‐assessments, strategies and tools for well‐being.
Managing the challenges of peer support roles: boundaries, burnout, compassion fatigue, working with distressed and suicidal people.
Compassion‐focused approaches to peer support.
Practical self‐soothing techniques for well‐being.
Asset mapping of local support resources.
Building a peer supporter network of mutual support.

Many peer supporters had personal experiences with attempted suicide and clients with suicidal ideations, some of whom had taken their own lives. To reduce potential triggers, the programme included break‐out rooms and well‐being activities, such as Tai Chi, art‐based mindfulness exercises and the creation of personal grounding toolkits (items to stimulate the five senses based on dialectical behaviour therapy).

Upon completing the training, peer supporters were invited to participate in semi‐structured interviews to explore their experiences; these findings are reported in this article. A constructivist epistemology [35] guided this exploration, focusing on participants' construction of meaning and their unique experiences throughout the co‐produced training.

### 2.2 Participants and Recruitment

The project consisted of two phases. In Phase 1, the research team engaged peer support organisations in North East England to co‐produce an emotional well‐being training programme through interviews. During these interviews, participants discussed their experiences as peer supporters, including benefits, challenges and unmet training and emotional well‐being needs. They were also asked for input on training topics they felt should be included. These data were thematically analysed [36] and used to design a training programme tailored to their needs. To ensure alignment with these needs, the proposed topics were presented back to the peer supporters.

Phase 2 comprised three stages: recruitment, training and post‐training interviews. Initially, participants from Phase 1 were invited to attend the training. Toward the end of the training, all participants, both in‐person and online, were invited to participate in a short interview to discuss their experiences. A purposeful sampling strategy was used, allowing participants to opt into each subsequent stage of the project. Snowball sampling was also employed, with participants informing their networks about the opportunity to participate in Phase 1 interviews, Phase 2 training and post‐training interviews.

Ten participants (five females and five males) from seven local organisations took part in post‐training interviews (see Table 2 for pseudonyms and settings). All participants worked in the third sector as peer supporters, with only one being paid for their work. Peer supporters' roles centred on guiding others through their journey to recovery. Their roles primarily involved guiding clients through their recovery journeys. They were assigned clients by their organisations, most of whom were early in recovery and experiencing significant challenges. While responsibilities varied, they generally focused on supporting clients with recovery, discussing triggers, signposting to services and sharing their own recovery experiences to offer empathetic support.

**Table 2:** Peer supporters and their respective organisation.

Participant pseudonym	Peer support setting
Yusuf	Addiction recovery
Sarah	Addiction recovery
Mariam	Supporting homeless people
Zayn	Men's mental health support
Debra	Addiction recovery
Marta	Mental health support
Jeff	Multicultural organisation
Alan	Veteran support
Zahra	Multicultural organisation
Alex	Mental health support

### 2.3 Data Collection

We sought to explore peer supporters' experiences with a co‐produced training programme that addressed their unmet training needs. Specifically, we focused on identifying the most beneficial elements of the programme for enhancing their knowledge and skills, as well as the perceived effectiveness of practical self‐care techniques. A semi‐structured interview schedule was used to collect data, with questions agreed upon by the research team.

In line with the research aims, the interviews included open questions about training experiences and thoughts on developing future sessions and a peer supporter network. The selection of topics was underpinned by previous literature and Phase 1 of the project. The schedule was used flexibly, with follow‐up questions to explore responses in detail. Peer supporters gave verbal consent and were individually interviewed by the first author. Interviews were conducted via Microsoft Teams (*n* = 9) and telephone (*n* = 1), lasting between 9.43 and 25.31 min (*M* = 16.81; standard deviation = 6.29), and were transcribed verbatim. All identifying information was anonymised using pseudonyms to maintain privacy.

No financial incentives were provided. This small‐scale training programme focused on understanding the training and well‐being needs of peer supporters in North East England (Phase 1; reported elsewhere) and exploring their experiences with the co‐produced training programme (Phase 2). Interviews ceased when the research team determined sufficient data had been collected to meet the project's aims, as saturation is not suitable for a reflexive thematic analysis approach [37].

### 2.4 Analytic Approach and Data Analysis

A constructivist epistemology guided this qualitative research, an approach to exploring peer supporters' experiences that has been used previously [38]. This position assumes no single objective reality, as knowledge is constructed individually, which facilitated peer supporters in sharing their unique experiences of the co‐produced training programme.

We employed a reflexive approach to thematic analysis, ensuring the analysis remained grounded in the data [39], and respected participants' individual experiences. A latent coding approach enabled a deeper understanding of participants' experiences [36]. Using an inductive approach, we focused on understanding the meaning constructed by participants within their social reality. Although the research team was aware that data would relate to the training topics, our epistemological stance prioritised participants' individual perspectives. Thus, an inductive approach was most suitable.

Thematic analysis was used to examine the data following Braun and Clarke's [40] six‐stage analysis: (1) the researchers familiarised themselves with the whole data set via reading and re‐reading of the transcripts; (2) the researchers generated initial codes; (3) the research team then searched for themes within the initial codes, gathering data relevant to initial themes; (4) themes were then reviewed on two levels, Level 1 comprised checking themes congruent with extracted data and Level 2 comprised checking themes were congruent with the whole data set; (5) themes were then defined and named, and became more specific, refined and detailed; and (6) findings were then written up. Several analysts were utilised to code the data set and refine themes and subsequent data extracted. Three analysts coded each transcript (Laura Kane, Lauren Walker and Robert M. Portman), with coding conducted blind. Once all analysts had completed their initial coding and collated these codes into potential themes, a research meeting was held and each analyst presented their findings. This approach enabled the research team to gain a deeper understanding of the data. Once consensus had been reached, the themes were constructed by LK, LW and RP, and presented at a team meeting. Themes were refined in line with the team's feedback. This collaborative approach enhanced confirmability and, thus, trustworthiness [41]. Finalised themes were reported once a consensus between all analysts had been reached. Several public contributors with lived experience or peer support experience helped develop the Phase 2 training and Phase 2 interview questions and provided reflections on the analysis.

### 2.5 Ethical Considerations

This study was granted ethical approval by the Teesside University research ethics committee.

## 3 Results

Thematic analysis of the data led to the construction of three primary themes: (1) *Increasing psychological preparedness and identifying self‐care and coping strategies*; (2) *It's good to know you're not alone*; (3) *Toward the future: next steps*.

### 3.1 Theme 1: Increasing Psychological Preparedness and Identifying Self‐Care and Coping Strategies

This theme highlights peer supporters' motivation to acquire new skills to enhance their roles and manage their own emotional well‐being. Overall, participants shared positive reflections on their training experience, particularly valuing the new knowledge that boosted their self‐confidence:

I learnt a lot. It helped a lot with, I think it was just more confidence building in myself. *Sarah (Female, addiction recovery)*

Sarah's comment highlights how the training increased her confidence as a peer supporter. Other participants also found specific topics valuable, such as handling client anger and applying coping strategies—skills particularly relevant to their work:

Yes, you know being aware of anxiety issues and anger issues, particularly in the work that we do with vulnerable people who don't feel safe, anger is a very easy emotion to access for them and I'll be able to use the techniques we learned in the peer support course to handle that side of things, you know. *Debra (Female, addiction recovery)*

Debra reflected on her experience and saw practical benefits from the training, including tools for managing client anger. Sensitive topics like suicide were also addressed, which was well‐received:

I really found the suicide training very interesting and very helpful. I think at times, that can be an area that is not missed, but isn't given as much as it could be. So, I really found that very useful. *Zayn (Male, men's mental health support)*

Zayn noted that training on suicide is often limited, despite its importance. To mitigate distress around this topic, the programme included regular breaks for relaxation activities:

The way that the suicide part of it was handled was excellent. You know lots of breaks and using self‐soothing techniques and you know the Tai Chi and things like that really soothed those trigger points that could have been there, you know. *Debra (Female, addiction recovery)*

This approach appears effective in balancing necessary training on sensitive topics with strategies to support emotional well‐being. The use of Tai Chi breaks, in particular, was valued and could be adopted in future training programmes to address sensitive subjects with care.

Participants also appreciated the training's emphasis on prioritising their own well‐being, a crucial reminder for those in support roles:

Most of all, it was the reminder that I've got to look after myself and I have got to put myself first. I had gone through strategies, before, with my own mental health but sometimes you just stop using them. You just fall into life, and that reminder was excellent, yes. *Alan (Male, veteran support)*

Because I think that can be kind of a big issue when you're in peer support, you forget about yourself a lot and it's really good to kind of be reminded to think about yourself a bit more. *Marta (Female, mental health support)*

Some participants reflected on how they planned to apply self‐care techniques learned during training, both personally and professionally:

I'll use, obviously, the self‐soothing techniques, but I'll also pass those on to clients as well you know, and help them use some of those techniques to help themselves, you know. *Debra (Female, addiction recovery)*

Alan expressed an interest in exploring more relaxation practices inspired by the training:

I took a year's subscription out on Head Space because I'm going to go back down the meditation route. I'm interested, now, as well, I had done yoga before, which it was a little bit like the tai‐chi with Suraj, and I found that excellent. *Alan (Male, veteran support)*

While Debra and Alan discussed future applications of their training, Marta reflected on the importance of avoiding burnout and decided to take a step back:

I'm, like, actually yes, the risk of burnout is real in peer supporters. People forget how kind of demanding it is sometimes I think and, yes, I don't want to get to that point of burnout. So, thinking about my emotional well‐being from this course is like, yes, I'm stepping back a little bit from things, I think. *Marta (Female, mental health support)*

In summary, this theme encapsulates the knowledge acquired by peer supporters as part of the training programme. Participants appreciated the inclusion of training on role‐specific elements such as suicide and emotional well‐being techniques, and they discussed the relevance and practical applications of these elements to their peer supporter roles.

### 3.2 Theme 2: “It's Good to Know You're Not Alone”

In this theme, participants identified the opportunity to network and connect with other peer supporters as a significant benefit of the training, which enhanced their sense of belonging. Many reflected on the pervasive social isolation in their roles but reported feeling less alone after the training.

It made me kind of feel less alone as a peer supporter, to kind of be able to speak to other people who do similar things to me, have kind of similar issues around it as well. *Marta (Female, mental health support)*

Similarly, Alan and Debra acknowledged the loneliness of their work and expressed how the training connected them with fellow peer mentors, strengthening their sense of connectedness:

I've got very little sort of support where I work, from a well‐being side of things, little to no support, actually. So that was a big help, yes. That was good. *Alan (Male, veteran support)*

Debra explained how meeting with peers served as a source of social support:

That it was good to meet up with your peers who work in that area, and you can let off a bit of steam, you know. It's good to know that you're not alone in it, I think. *Debra (Female, addiction recovery)*

Connecting with peers provided an outlet for sharing knowledge and experiences, which also helped relieve emotional distress. Participants valued the opportunity to offload stress by communicating with others who understood their challenges. Marta explained how it can be difficult for peer supporters to express their own struggles to those they support:

Like, if you're having a bad day you don't get to say you're having a bad day necessarily because you want to be kind of supportive and hopeful for those people you're helping. So, I think it is important to meet other peer supporters and be able to say, I'm struggling, can I have some support? Because they understand that kind of position and there's no one who's kind of above anybody else in that situation. *Marta (Female, mental health support)*

Marta described the value of accessing support from those perceived as equals and with similar experiences, valuing lived experience when accessing social support. Mariam also describes an example of this, and recounts discussing her own struggles with a peer supporter during the training, who had also recently lost their father:

Like, this week's a tough one because it's my dad's first memorial. And then I messaged somebody yesterday just to see how they were and found out that, like, their dad had just passed away last week. So, we've got that support network as well, for our own well‐being in personal lives as well now, which is pretty good. *Mariam (Female, supporting homeless people)*

Mariam highlighted how she benefitted from the mutual support between peer supporters, particularly when both peers had shared a lived experience. Mariam also discussed how she benefitted from peer supporters with expertise different to her own: *One of the other people who, his background's like with gambling, obviously I found out there's been an issue, there's somebody in the family with that, so they're helping me with that as well.* Here, Mariam illustrated how the training programme was underpinned by the values of peer support, enabling participants to share their expertise with each other and offer guidance both professionally and personally.

In summary, in this theme, participants highlighted the training programme as a strength as it enabled peer supporters to gain support from their peers. As highlighted by some of the participants, working as a peer supporter can be emotionally taxing and equally lonely, and they have few opportunities to offload negative emotions or experiences. However, peer supporters describe how this training programme allowed them to interact with other peer supporters, share experiences and stories, and offer advice and support to each other. These findings emphasise the need to ensure that peer supporters' needs are met, to maintain their own well‐being, so that they are able to continue providing support for others.

### 3.3 Theme 3: Toward the Future: Next Steps

Participants expressed a strong motivation to establish a peer supporter network that would allow individuals from diverse backgrounds to share experiences, insights and mutual psychosocial support. This motivation was largely derived from their positive engagement with the training programme.

Yes, I think we've all got such a kind of wealth of knowledge that it's great to be able to kind of share that with each other and pick up all these, yes, resources that everybody else has got. *Marta (Female, mental health support)*

Similarly, Mariam expressed the value of working alongside peer supporters from different backgrounds:

Even, like, things from [Peer Support Organisation] … because they've lived on the streets, that's who we go out and help as well, for them to come and talk to us or even come out with us one night some of them, you know. *(Female, supporting homeless people)*

For Mariam, learning from other peer supporters expanded her ability to support diverse populations. The desire to provide person‐centred support led to a strong interest in a peer supporter network to further enhance knowledge. Marta emphasised that such a network could reduce burnout and improve retention among peer supporters:

Yes, there's definitely the scope for more opportunity to kind of get things out and I think you'd get more kind of long‐term peer supporters if that kind of network is in place. People wouldn't burn out, get compassion fatigue, move on to other things, they'd kind of stick with it a bit longer. *Marta (Female, mental health support)*

Marta saw the network not only as beneficial for her well‐being but also for clients, who could benefit from consistent support and a trusting relationship over time.

Beyond the network, participants were also eager to continue learning, especially on challenging topics that needed more depth in the training. For instance, Sarah discussed the importance of a deeper understanding of intense emotions like anger:

There should be a bit more on the anger because there's, like, the scenarios I think, people have like family members or something that have the anger in, like the anger side, dealing with anger a bit more. *Sarah (Female, addiction recovery)*

Sarah's reflection highlights the emotional labour involved in peer support, particularly when dealing with intense emotions that may also affect clients' families.

Mariam shared similar thoughts on topics that needed to be covered in more depth going forward:

I mean even some of the stuff that we did I think we could go back and revisit a bit more in‐depth. Because I know we skipped over, like, the self‐harm and the aggression bit, we didn't really cover any of that. So, I just think maybe some of the stuff to, you know, go back over and do it again. *Mariam (Female, supporting homeless people)*

Mariam reiterated the emotional labour inherent in peer support, and the value of focusing on difficult topics such as self‐harm and coping with aggressive behaviours.

In summary, this theme reveals participants' interest in a peer supporter network and ongoing training opportunities. The benefits of peer support during training inspired participants to propose a formal network to continue to support their well‐being. Additionally, the desire for more training on difficult topics reflects peer supporters' commitment to professional growth and their dedication to fulfilling their roles with the appropriate knowledge and skills.

## 4 Discussion

This study explored peer supporters' experiences of attending a bespoke co‐produced training programme, aimed at addressing unmet training needs and improving emotional well‐being. The thematic analysis produced three themes: (1) Increasing psychological preparedness and identifying self‐care and coping strategies; (2) “It's good to know you're not alone”; and (3) Toward the future: next steps.

The first theme, ‘Increasing psychological preparedness and identifying self‐care and coping strategies’, reflects participants' appreciation for training on challenging aspects of peer support, such as managing client aggression and establishing boundaries, along with learning practical techniques for self‐care and well‐being maintenance. In the second theme, ‘It's good to know you're not alone’, peer supporters reflected on the benefits of connecting with others during training. They acknowledged the social isolation of their roles and realised the importance of social support for their emotional well‐being. The third theme, ‘Toward the future: next steps’, captured participants' desire to build a mutual support network for knowledge exchange and support. They expressed a commitment to ongoing learning, particularly on more distressing and complex topics.

Overall, the findings indicate an overwhelmingly positive training experience, highlighting three key discussion points: (1) the role of co‐production in addressing unmet training needs, (2) the value of peer support among peer supporters and (3) the importance of delivering training with sensitivity.

Phase 1 of this project [29] involved interviews with peer supporters from diverse backgrounds, both formal and informal, statutory and third‐sector roles. These covered areas such as addiction recovery, domestic and sexual violence, refugee and asylum support, mental health, LGBTQ+ and veteran support. The research team examined the training and well‐being needs of peer supporters, exploring both the strengths and challenges of various peer support contexts. From this research, three key themes emerged: lack of training and support, role ambiguity and emotional labour. Training was often insufficient or unavailable, leaving peer supporters unprepared. Many experienced triggers and stressors that disrupted their recovery, compounded by inadequate support. Role ambiguity posed challenges for implementing boundaries and managing self‐disclosure, leading to emotional distress, exhaustion and burnout. Although peer supporters often noted the benefits of their role for their own recovery, finding meaning and purpose in helping others, many of their training and well‐being needs remained unmet.

This study illustrates the importance of co‐producing peer supporter training, as previously suggested [16]. Prior literature has documented the impacts and consequences of peer supporter training programmes [24, 25, 26, 28]. Mindful of potential negative effects, the present study focused on maintaining peer supporters' well‐being throughout the training and their ongoing roles. Phase 1 attested to the adverse consequences experienced by peer supporters, especially when little or no training or support was available [29]. Participants valued the inclusion of well‐being strategies, particularly when discussing sensitive topics such as self‐harm and suicide, given their personal and professional encounters with these [29]. By exploring co‐produced training, this study emphasises the need to prioritise peer supporters' well‐being and offers insights for both statutory and third‐sector organisations on promoting wellness from training to practice.

Peer supporters discussed their positive experiences of the role‐specific training and reflected on which aspects of the training they valued most. They regarded these aspects as crucial for building confidence in their roles. Participants noted that such topics are rarely covered in typical training sessions; this aligns with existing literature showing a gap in role‐specific training opportunities for peer supporters [16, 21], which can reduce role uptake and maintenance of peer support roles [9].

The lack of training on critical issues like suicide represents a significant disadvantage for the peer support workforce, especially considering the high likelihood that they will encounter individuals at risk of suicide, given the nature of their work. Some participants also indicated that due to the limited timeframe of the programme, certain topics could have been explored in greater depth. Despite this, there was a strong interest in establishing a peer support network. Such a network would enable more comprehensive training on complex topics and provide a pathway for ongoing professional development. The findings reinforce the need to train peer supporters on aspects of their roles where they feel underprepared, as this can boost their confidence, enhance their effectiveness and support role sustainability.

Previous literature has highlighted a lack of standardised peer support training, evidenced by the heterogeneity of existing programmes [16, 17]. This inconsistency means that peer support training programmes differ significantly from one another, meaning that essential topics are not consistently or thoroughly covered across programmes. Peer supporters have expressed a need for training on specific issues such as self‐disclosures, boundaries, peer relationships and managing overwhelming emotions [21, 42]. In the current study, topics were co‐produced with academic researchers to align with peer supporters' needs, addressing limitations seen in previous training programmes. For example, Simpson et al. [26] identified that emotional attachments between peer mentors and clients are often overlooked, leaving peer mentors feeling unprepared for the emotional labour involved. To address this, the bespoke programme included training on emotional attachments, which is linked to reduced burnout risk [19].

The positive feedback from participants, outlined in Theme 1, supports the need for training, that is congruent with the essence of peer support [17, 19], which can be ensured through co‐production [16].

The second key finding is the social isolation commonly experienced by peer supporters. Participants described feeling alone in their roles, unable to share their mental strain to avoid burdening others. When coupled with emotional labour, this social isolation increases the risk of burnout among peer supporters [20]. Given the emotionally demanding nature of their work, it is pertinent to reduce burnout risk by bolstering social support. However, the academic literature indicates that peer supporters receive inadequate social support and supervision [18, 42], a sentiment echoed by our participants, who valued the chance to connect and network with other peer supporters. This finding aligns with previous studies documenting the significant educational and emotional benefits of peer support [12, 23, 26]. Like these studies, our findings show that reduced perceived loneliness and improved well‐being can result from peer mentorship.

As a result of their training experience, peer mentors identified a need for a local network to facilitate ongoing knowledge sharing and psychosocial support, a benefit also noted by Moran et al. [12]. Upon completing the training, participants expressed a strong motivation to continue their professional development, showing enthusiasm for gaining a deeper understanding of complex topics covered during the programme. They also saw the potential of a local peer mentor support network to meet their future training needs effectively.

Our findings highlight best practices for the sensitive delivery of peer support training. Maintaining peer supporters' well‐being during and after training has been identified as a challenge in previous research [25, 26, 28]. Participants in the present study admitted to neglecting their own emotional well‐being and mental health, reporting heightened stress, emotional exhaustion and burnout—consistent with prior findings [19, 20].

Moran et al. [19] and Repper and Watson [23] recommend incorporating therapeutic skills into peer supporter training. In this study, participants valued the inclusion of relaxation activities and well‐being strategies, especially when addressing sensitive topics such as suicide. They reflected on the positive impact these techniques had on their well‐being and expressed interest in using them, both personally and professionally. We highlight this approach as good practice and recommend that future training programmes incorporate relaxation exercises to help mitigate negative emotions when dealing with challenging content.

### 4.1 Strengths and Limitations

Prior studies have documented the adverse impacts of peer support training, but the positive aspects of modifying training programmes to enhance participant well‐being have not yet been explored. By integrating well‐being activities and considering the lived experiences of peer supporters, this study provides new and important insights into how training can be both effective and supportive, informing future programme designs to better support peer supporters.

A limitation of this study is the short duration of the interviews which may have stemmed from participants scheduling their involvement during brief periods to accommodate their work schedules, potentially limiting the detail obtained. Mindful of not overburdening the peer supporters, the team deliberately focused the questions on their personal experiences of the training.

### 4.2 Recommendations and Future Directions

Our findings highlight the importance of co‐producing training programmes together with peer supporters to ensure their training and well‐being needs are met. This training programme was conducted on a small scale with time constraints. Future studies could replicate this programme with a larger sample size and over a longer duration.

As previously noted, prior research has evidenced the negative impact training can have on peer supporters' mental health and recovery [25, 28]. Based on our findings, we recommend that future peer mentor training is co‐produced and places peer supporters' emotional well‐being and mental health at the heart of the programme. This approach should involve the active participation of peer supporters in designing training materials and methods to ensure they are both effective and supportive. Moreover, regular assessments should be incorporated to monitor and address any emotional challenges that arise during the training process.

Furthermore, future studies could explore how peer support training varies between groups, such as those focusing on mental health versus marginalised populations. Additionally, demographic characteristics of peer supporters should be considered in future research to explore how training could be tailored to further enhance its effectiveness. Such research could reveal important differences and help improve training effectiveness for diverse communities.

Peer supporters in this study reported on the need for a peer mentoring network for peer supporters to reduce isolation and emotional distress and to concurrently increase knowledge and insight through shared knowledge and experiences. Future research should explore the barriers and facilitators of the development and maintenance of a peer mentoring network for peer supporters. Additionally, research could consider the impact of digital platforms on expanding access and engagement within the network, ensuring that it reaches a diverse group of peer supporters across different regions and contexts.

This study employed an inductive approach, intentionally foregoing the application of existing theoretical frameworks to allow for an unbiased exploration of emerging themes. While this allowed us to develop a grounded understanding of the specific phenomena related to peer support training as experienced by the participants, integrating theoretical perspectives could contribute to a more robust theoretical grounding of the results. This might be an aspect for future studies to consider.

## 5 Conclusion

The present study contributes to the expanding body of literature on effective peer supporter training and underscores the importance of addressing supporters' psychosocial needs for both their roles and personal well‐being. Training programmes should be highly accessible, designed with consideration for peer supporters' lived experiences, and aligned with the core principles of peer support through co‐production. They should also foster a collaborative environment where participants can learn from each other and develop collectively, a method that has proven beneficial in this context.

## Author Contributions

**Laura Kane:** conceptualisation, writing–original draft, formal analysis, writing–review and editing, methodology, project administration. **Lauren Walker:** conceptualisation, writing–review and editing, funding acquisition, formal analysis. **Judith Eberhardt:** writing–review and editing, supervision. **Robert M. Portman:** conceptualisation, writing–review and editing, funding acquisition. **Emma‐Lily Proctor:** conceptualisation, methodology. **Hannah Poulter:** conceptualization, funding acquisition, writing–review and editing, methodology, supervision. **Catherine O'Neill:** conceptualisation, funding acquisition, writing–review and editing, methodology, project administration, supervision.

## Ethics Statement

Our study was approved by the Teesside University research ethics committee (reference no. 2022 Jan 7693 Poulter).

## Consent

All participants provided written informed consent before enrolment in the study.

## Conflicts of Interest

The authors declare no conflicts of interest.

## Data Availability

The data that support the findings of this study are available on request from the corresponding author. The data are not publicly available due to privacy or ethical restrictions.
